# 

**Published:** 1870-04

**Authors:** 


### *American Medical Association.*

Office of Permanent Secretary—Wm. B. Atkinson, M.D., 1400 Pine st., S.W. cor. Broad, Philadelphia.

The twenty-first annual session will be held in Washington, D.C., May 3, 1870, at 11 A.M. The following committees are expected to report:

On Cultivation of the Cinchona Tree—Dr. Lemuel J. Deal, Pennsylvania, chairman. On the Cryptogamic Origin of Disease, with special reference to Recent Microscopic Investigations on that subject—Dr. Edward Curtis, U.S A., chairman. On the Doctrine of Force, Physical and Vital—Dr. John Waters, Missouri, chairman. On Variola—Dr. Joseph Jones, Louisiana, chairman. On the Relative Advantages of Syme's and Pirogoff's mode of Amputating the Ankle—Dr. G. A. Otis, U.S.A., chairman. On a National Medical School—Dr. F. G. Smith, Pennsylvania, chairman. On Commissioners to aid in Trials involving Scientific Testimony—Dr. John Ordronaux, N.Y., chairman. On the Climatology and Epidemics of Maine, Dr. J. C. Weston; New Hampshire, Dr. P. A. Stackpole; Vermont, Dr. Henry Janes; Massachusetts, Dr. H. I. Bowditch; Rhode Island, Dr. C. W. Parsons; Connecticut, Dr. E. K. Hunt; New York, Dr. W. F. Thoms; New Jersey, Dr. Ezra M. Hunt; Pennsylvania, Dr. D. F. Condie; Maryland, Dr. O. S. Mahon; Georgia, Dr. Juriah Harriss; Missouri, Dr. Geo. Engleman; Alabama, Dr. R. F. Michel; Texas, Dr. T. J. Heard; Illinois, Dr. R. C. Hamil; Indiana, Dr. J. F. Hibberd; District of Columbia, Dr. T. Antisell; Iowa, Dr. J. C. Hughes; Michigan, Dr. Abm. Sager; Ohio, Dr. T. L Neal; California, Dr. F. W. Hatch; Tennessee, Dr. B. W. Avent; West Virginia, Dr. E. A. Hildreth; Minnesota, Dr. Samuel Willey; Virginia, Dr. W. O. Owen; Delaware, Dr. L. B. Bush; Arkansas, Dr. G. W. Lawrence; Mississippi, Dr. W. Compton; Louisiana, Dr. L. T. Pimm; Wisconsin, Dr. J. K. Bartlett; Kentucky, Dr. J. D. Jackson. On Veterinary Colleges—Dr. Thomas Antisell, D. C., chairman. On Medical Ethics—Dr. Lewis A. Sayre, N.Y., chairman. On American Medical Necrology—Dr. C. C. Cox, Maryland, chairman. To Memoralize State Medical Societies—Dr. N. S. Davis, Illinois, chairman. On Nomenclature of Diseases—Dr. F. G. Smith, Pennsylvania, chairman. On Medical Education—Dr. T. G. Richardson, Louisiana, chairman. On Medical Literature—Dr. J. J. Woodward, U.S.A., chairman. On Prize Essays—Dr. Grafton Tyler, D. C., chairman.

Voluntary communications will be presented by Dr. John Curwen, Pennsylvania, on the Proper Treatment of the Insane; Dr. Nathan Allen, Massachusetts, on the Physiological Laws of Human Increase.

Secretaries of all medical organizations are requested to forward lists of their delegates as soon as elected, to the Permanent Secretary.

Any respectable physician who may desire to attend, but cannot do so as a delegate, may be made a *member by invitation*, upon the recommendation of the Committee of Arrangements. W. B. Atkinson.

